# Abstracts

**DOI:** 10.1155/S1463924683000437

**Published:** 1983


Journal of Automatic Chemistry, Volume 5, Number 4 (October–December 1983), pages 170–173

### Abstracts

### Page 174

### A potentiometric analyser based on the ZX81 microcomputer

S. W. Bateson *et al.**

A ZX81 microcomputer has been combined with a high-precision interface system to control a potentiometric titration assembly. This has been programmed for a variety of options including the derivation of electrode response calibrations. Illustrative results are given for precipitation titration of silver nitrate with potassium bromide.

### Page 182

### An evaluation of the AM 721 ion-selective electrode system for the estimation of sodium and potassium in plasma, urine and whole blood

P. West

An evaluation is described of the AM 721 ion-selective electrode system (Kontron Instruments Ltd) for the measurement of sodium and potassium in plasma, urine and whole blood. There was good agreement between results obtained with this instrument and routine flame photometry in terms of precision and accuracy for both plasma and urine. The ease and speed of operation of the AM 721, together with its small size and the need for as little as 50 $\mu$l of plasma or urine, makes it a useful instrument. In addition, the close agreement between values in whole blood and plasma makes it ideal in units where a centrifuge may not always be available and results needed urgently.

### Evaluation du système AM 721 pour l'analyse du sodium et potassium dans le plasma, l'urine et le sang par électrode ion-sélective

P. West

Une évaluation du système AM 721 par électrode ion sélective (Kontron Instruments Ltd) pour la mesure du sodium et du potassium dans le plasma, l'urine et le sang est décrite. Une bonne corrélation entre les résultats obtenus par cet instrument et la photométrie de flamme de routine a été trouvée pour le plasma et l'urine. La facilité et la vitesse d'opération du AM 721 en plus de sa petite taille et l'utilisation d'un trés petit échantillon de 50 $\mu$l de plasma ou d'urine en font un instrument très pratique. En plus, la bonne correspondance entre les valeurs dans le sang et le plasma en font un instrument idéal lorsqu'une centrifuge n'est pas accessible ou que les résultats sont demandés d'urgence.

### Eine Evaluation des ionenselektiven Elektrodensystems AM 721 für die Bestimmung von Natrium und Kalium in Plasma, Urin und Gesamtblut

P. West

Die Arbeit beschreibt eine Evaluation des ionenselektiven Elektrodensystems AM 721 (Kontron Instruments Ltd) für die Bestimmung von Natrium und Kalium in Plasma, Urin und Gesamtblut. Für die Resultate, die mit diesem Instrument und mit Routine-Flammenphotometrie erhalten wurden, bestand gute Übereinstimmung in bezug auf Präzision als auch Genauigkeit bei Messungen am Plasma und Urin.

Die leichte Bedienbarkeit und die Geschwindigkeit des Betriebs des AM 721 zusammen mit der Kompaktheit des Designs und dem geringen Bedarf von bis zu nur 50 $\mu$l Plasma oder Urin, machen dieses System zu einem nützlichen Instrument. Die enge Übereinstimmung der Werte für Gesamtblut und Plasma ist zusätzlich ideal in Verhältnissen, wo eine Zentrifuge nicht zur Verfügung steht, aber Resultate dringend benötigt werden.

### Page 188

### Analytical and economical optimization of a glucose method with immobilized enzymes

I. Andersen and S. Hannibal

A glucose method, SE 40046FD9 (Technicon), using immobilized enzymes in an air-segmented continuous-flow system has been modified and optimized.

Uncentrifuged whole blood or urine is used instead of serum. The rate of determination is enhanced from 60 to 100/h. Carry-over is less than 3·0%. Precision (within series) is 2·0%.

Durability of the immobilized enzymes is three months instead of one month. Calibration function is linear to an upper limit between 20 and 40 mmol/l depending on the age of the enzyme reactor. Reagent consumption is reduced in different ways depending on the analytical system, i.e. whether the analyser consists of one or several channels. Solenoid valves protect the enzyme coil during the multichannel system cleaning. Comparison of the results on whole blood/urine using this modified Immobilized Hexokinase-Glucose-6-P-Dehydrogenase method and the Glucose Dehydrogenase method shows excellent agreement.

### Optimisation analytique et économique d'une méthode pour glucose par enzymes immobilisés

I. Andersen and S. Hannibal

Une méthode pour glucose (SE 40046FD9 Technicon) utilisant des enzymes immobilisés en un système à flux continu par ségmentation avec air a été modifiée et optimisée. Du sang non-centrifugé ou de l'urine est utilisé à la place du sérum. Le nombre de déterminations est augmenté de 60 à 100 par heure. La contamination est inférieure à 3%, la précision (entre séries) est de 2%.

La durée de vie des enzymes immobilisés est de 3 mois au lieu d'un mois. La fonction de calibrage est linéaire jusqu'à une limite supérieure entre 20 et 40 mmol/l dépendant de l'age du réacteur à enzymes. La consommation de réactif est diminuée de façons différentes dépendant du système analytique, c'est-à-dire si l'analyseur consiste d'un ou de plusieurs canaux. Des vannes sélénoides protègent la spirale à enzyme pendant le nettoyage du système multicanaux. La comparaison des résultats du sang complet/urine utilisant cette méthode modifiée avec hexokinase-glucose-6-P-dehydrogénase immobilisé et la déhydrogénase de glucose montre un excellent accord entre les deux méthodes.

### Analytische und wirtschaftliche Optimierung einer Glucose-Methode mit immobilisierten Enzymen

I. Andersen and S. Hannibal

Eine Glucose-Methode, SE40046FD9 (Technicon), die immobilisierte Enzyme in einem mit Luftblasen segmentierten kontinuierlichem Durchflusssystem benützt, wurde modifiziert und optimiert.

Statt Serum wurde unzentrifugiertes Gesamtblut oder Urin benützt. Der Bestimmungsdurchsatz wurde von 60

* German and French summaries will be published in the January–March issue.

auf 100 pro Stunde erhöht. Die Probenverschleppung beträgt weniger als 3%. Die Präzision (innerhalb einer Serie) beträgt 2%.

Die Benützungsdauer der immobilisierten Enzyme beträgt drei Monate statt nur einem Monat. Die Kalibrationsfunktion ist bis zu einer oberen Grenze zwischen 20 und 40 mmol/l, abhängig vom Alter des Enzymreaktors, linear. Der Reagensverbrauch ist je nach analytischem System auf verschiedene Art vermindert worden, das heisst der Analysator besteht aus einem oder mehreren Kanälen. Magnetventile schützen die Enzymspirale während des Waschzyklus im Vielkanal-System. Ein Vergleich der Resultate für Gesamtblut/Urin unter Benützung dieser modifizierten immobilisierten Hexokinase-Glucose-6-P-Dehydrogenasemethode und der Glucose-Dehydrogenasemethode zeigen beste Übereinstimmung.

---

### Page 193

### Ultra-trace level detection of arsenic and selenium using a commercially available hydride generator with atomic-absorption detection

R. W. Ward and P. B. Stockwell

Many papers have been published on the analysis of arsenic and selenium by hydride generation-atomic spectroscopy. A variety of reduction cells and detection systems have been proposed. The virtues of batch and continuous-flow hydride generation have also been argued. This work will show that by careful design a commercial continuous-flow hydride generator can produce a precise and sensitive system for the detection of metalloid elements. Detection levels for arsenic and selenium obtained were 0·04 and 0·07 ppb respectively. In addition the system is simple to operate and allows large sample throughputs, up to 40 samples/h.

### Détection d'ultra-traces d'arsénic et de sélénium utilisant un générateur d'hydride commercial avec détection par absorption atomique

R. W. Ward and P. B. Stockwell

Plusieurs publications décrivent l'analyse de l'arsénic et du sélénium par génération d'hydride/spectroscopie par absorption atomique. Une grande variété de cellules de réduction et de systèmes de détection ont été proposés. Les avantages de la génération d'hydride en continu ou en discontinu ont été discutés. Ce travail va montrer qu'une construction judicieuse d'un système continu commercial pour la génération d'hydride peut produire un système précis et sensible pour la détection des éléments métalloïdes. Les niveaux de détection pour l'arsénic et le sélénium obtenus étaient de 0·04 et 0·07 ppb respectivement. En plus, le système est facile à opérer et permet de traiter jusqu'à 40 échantillons par heure.

### Ultra-Spurenbestimmung von Arsen und Selen mit einem kommerziell erhältlichen Hydride-Generator mit Atomabsorptionsdetektion

R. W. Ward and P. B. Stockwell

Zum Thema der Analyse von Arsen und Selen mit Hilfe der Kombination Hydride-Generator/Atomabsorptions-Spektroskopie wurden bereits viele Arbeiten publiziert. Eine Anzahl von Reduktionszellen und Detektionssystemen sind vorgeschlagen worden. Die Vorteile der kontinuierlichen und diskontinuierlichen Hydride-erzeugung wurden angeführt. Die vorliegende Arbeit zeigt, dass bei sorgfältiger Konstruktion mit einem kommerziellen, kontinuierlichen Hydride-Generator ein präzises und empfindliches System für die Detektion der metalloiden Elemente entstehen kann. Die erhaltenen Detektionsgrenzen für Arsen und Selen betrugen 0,04 bzw. 0,07 ppb. Das System ist zusätzlich einfach zu bedienen und erlaubt einen hohen Durchsatz von bis zu 40 Proben/Stunde.

---

### Page 197

### A microprocessor-controlled film balance system

R. Urhahn *et al.*

Design, performance and test data of a microprocessor-controlled film balance system are presented. The system consists of a control unit and a stepper-motor-driven spreading device which are connected to a conventional film balance. The main advantages of the system are efficient control of experiments by flexible measuring programs, precise automatic delivery of spreading solutions to the surface at different flow rates and facilitation of individual data analysis. With a little modification the control unit can be adapted to other film balances.

### Un système de balance pour couches minces controlé par microprocesseurs

R. Urhahn *et al.*

La construction, la performance et les résultats de tests d'un système ce balance pour couches minces contrôlé par microprocesseurs sont présentés. Le système consiste d'une unité de contrôle et d'un module de déposition activé par un moteur pas à pas qui sont connectés à une balance pour couches minces conventionnelle. Les avantages principaux de ce système sont le contrôle effectif des expériences par des programmes de mesure flexibles, le débit précis automatique des solutions de déposition sur la surface à différentes vitesses de débit et la simplification de l'analyse des données individuelles. Avec peu de modifications l'unité de contrôle peut être adaptée à d'autres balances pour couches minces.

### Dünnfilm-Wägesystem mit Mikroprozessorsteuerung

R. Urhahn *et al.*

Es werden Konstruktion, Leistungsdaten und Prüfresultate eines Mikroprozessor-gesteuerten Dünnschicht-Wägesystems präsentiert. Das System besteht aus einer Steuereinheit und einem Auftragungsgerät mit Schrittmotor, welche mit einer konventionellen Dünnfilm-Waage verbunden sind. Die Hauptvorteile des Systems bestehen in der effizienten Steuerung der Experimente mit Hilfe flexibler Messprogramme, präziser und automatischer Dosierung der Auftragungslösungen auf die Oberfläche mit verschiedenen Geschwindigkeiten, und der Ermöglichung individueller Datenanalyse. Mit wenigen Änderungen kann die Steuereinheit auch an andere Dünnfilm-Waagen angepasst werden.

---

### Page 201

### Six years' experience of in-house maintenance of clinical chemistry autoanalysers

D. J. Wyper *et al.*

The progress over the past six years of an in-house maintenance scheme for clinical chemistry autoanalysers is described. The scheme involves six Area Health Boards and is fully costed to enable comparison to be made with commercial alternatives.

It is shown to be of considerable financial benefit to the Health Boards concerned, as well as improving the technical expertise within the National Health Service. Consideration has to be given, however, to the volume of work and to the geographical distribution of the laboratories involved.

### 6 années d'expérience dans l'entretien interne d'auto-analyseurs pour la chimie clinique
D. J. Wyper *et al.*

Le progrès des dernières 6 années dans l'entretien interne des auto-analyseurs dans la chimie clinique est décrit. La disposition comprend 6 offices de santé régionaux et est accompagnée de calculations de frais pour permettre une comparaison avec des alternatives commerciales.

Il a été prouvé une amélioration financière considérable pour les autorités concernées et du progrès dans les connaissances techniques de l'Office National de Santé. Cependant, il faut considérer le volume du travail et la distribution géographique des laboratoires incorporés.

### Sechs Jahre Erfahrung im selbstdurchgeführten Unterhalt an Auto-Analysers für die klinische Chemie

D. J. Wyper *et al.*

Die Arbeit beschreibt den Fortschritt über die letzten sechs Jahre beim selbstdurchgeführten Unterhalt für Auto-Analysers der klinischen Chemie. Das Unterhaltsschema umfasst sechs Area Health Boards und basiert auf einer Vollkostenrechnung, um den Vergleich mit kommerziellen Alternativen zu ermöglichen.

Es wird gezeigt, dass beträchtliche finanzielle Vorteile für die Health Boards resultieren, aber dass auch die technische Expertise innerhalb des National Health Service verbessert werden. Jedoch ist der Arbeitsumfang sowie die geographische Verteilung der betroffenen Laboratorien zu berücksichtigen.

---

### Page 204

### Two years' experience with Motorola Micromodules as an interface for a flame photometer
R. A. Lutz

An example of interfacing a laboratory instrument to a minicomputer using commercially available micromodules is described. The system stores results in a memory with battery back-up. When the results are ready the microprocessor transmits the data automatically if the main laboratory minicomputer is running. The serum number and additional information has to be entered by the laboratory technician. If the minicomputer is turned off or not ready, the microprocessor keeps the results in its memory until data transfer is possible. This system is universally applicable and can be easily modified to any digital laboratory instrument. The system has now been connected for over two years to an IL Flame Photometer 343 and has proved itself reliable. Other microprocessors can also be linked to it by simple addition of corresponding boards. Only one line to the minicomputer is needed for up to eight instruments.

### 2 ans d'expérience avec des micromodules Motorola comme interface pour un photomètre de flamme
R. A. Lutz

Un exemple d'interface d'un instrument de laboratoire à un miniordinateur utilisant des micromodules commerciaux est décrit. Le système mémorise des résultats dans une mémoire soutenue par batteries. Quand les résultats sont prêts, le microprocesseur transmet les données automatiquement, si le miniordinateur de laboratoire principal est en fonction. Le numéro du sérum et d'autres informations doivent être introduits par le technicien de laboratoire. Si l'ordinateur n'est pas prêt ou hors fonction, le microprocesseur garde les résultats dans sa mémoire jusqu'à ce que le transfert de données devient possible. Ce système est applicable de façon universelle et peut facilement être modifié pour n'importe quel instrument de laboratoire digital. Ce système a été connecté pendant plus de 2 ans à un photomètre de flamme IL 343 et a travaillé de façon fiable. D'autres microprocesseurs peuvent également être connectés par simple addition des circuits correspondants et une seule ligne est nécessaire pour la connection du miniordinateur avec 8 instruments au maximum.

### Zwei Jahre Erfahrung mit den Motorola Mikromodulen im Einsatz als Interface für ein Flammenphotometer
R. A. Lutz

Die Arbeit beschreibt ein Beispiel für die Verbindung von einem Labormessgerät mit einem Minicomputer unter Benützung kommerziell erhältlicher Mikromodule. Das System speichert die Resultate in einem Speicher mit Batteriezusatz. Wenn die Resultate bereitstehen, übermittelt der Mikroprozessor die Daten automatisch, sobald der Labor-Minicomputer in Betrieb steht. Die Serum-Identifikation sowie zusätzliche Informationen müssen vom Benützer eingegeben werden. Wenn der Minicomputer nicht in Betrieb ist oder nicht bereitsteht, behält der Mikroprozessor die Daten in seinem Speicher bis eine Übermittlung möglich ist. Das System ist universell bis eine Übermittlung möglich ist. Das System ist universell verwendbar und kann leicht an irgendein digitales Labormessgerät angepasst werden. Das System ist bis jetzt seit über zwei Jahren an ein IL Flammenphotometer 343 angeschlossen und arbeitete zuverlässig. Weitere Mikroprozessoren können ebenfalls angeschlossen werden durch einfaches Einschieben entsprechender gedruckter Schaltungen. Für die Verbindung zum Minicomputer wird für acht Instrumente lediglich eine Leitung benötigt.

---

### Page 207

### Automated determination of zinc and copper in plasma
B. Sampson

The continuous-flow atomic absorption determination of copper and zinc in plasma or serum is described. n-Butanol is used as a diluent. The coefficient of variation achieved is less than 3%. Sample consumption is 200 $\mu l$. The optimum sample for analysis has been investigated, and a rapid increase in zinc content in the plasma obtained from heparinized blood has been found at room temperature. It is recommended that samples be kept cold and the plasma be separated as soon as is possible. No such effect is found for copper. A normal range for plasma copper and zinc is given.

### Détermination automatique du zinc et du cuivre dans le plasma
B. Sampson

La détermination du cuivre et du zinc dans le plasma ou le sérum par absorption atomique à flux continu est décrite. Le n-butanol est utilisé comme diluant. Le coéfficient de variation obtenu est inférieur à 3%. La consommation d'échantillon est de 200 $\mu l$. Les meilleurs échantillons pour l'analyse ont été étudiés et une augmentation rapide dans le contenu de zinc dans le plasma obtenu de sang héparinisé a été trouvé à température ambiante. Il est recommandé de garder les échantillons au frais et de séparer le plasma aussi rapidement que possible. Pour le cuivre aucun effet de ce genre a été trouvé. La plage normale pour le cuivre et le zinc dans le plasma est indiqué.

**Die automatisierte Bestimmung von Zink und Kupfer in Plasma**
B. Sampson

Die Arbeit beschreibt die Bestimmung von Kupfer und Zink in Plasma oder Serum mit Atomabsorbtion im Durchflussverfahren. Als Verdünnungsmittel wird n-Butanol benützt. Der erreichte Variationskoeffizient beträgt weniger als 3%. Der Probenverbrauch beträgt 200 $\mu$l. Die für die Analyse optimale Probe wurde untersucht, und es wurde bei Zimmertemperatur ein rascher Anstieg des Zinkgehaltes in Plasma gefunden, das von heparinisiertem Blut stammt. Es wird empfohlen, dass die Proben kühl gehalten werden und dass das Plasma so schnell als möglich absepariert wird. Kein solcher Effekt wurde für Kupfer beobachtet. Die Normalbereiche für Kupfer und Zink in Plasma werden angeführt.

---

### Page 210

**Cost-benefit parameters in evaluating automation**
T. M. Craig

This is one of the papers read at the symposium on cost analysis held on 22 April 1982 during the First International Congress on Automation in Clinical Chemistry (Barcelona, Spain)—the others were published in *Journal of Automatic Chemistry*, Vol. 5, No. 2.

The author presents useful financial parameters for evaluating the impact of automation in the clinical laboratory, plus some insight into the potential effect of automation on the hospital system.

**Les paramètres frais-bénéfice pour l'évaluation de l'automation**

T. M. Craig

Ceci est un des exposés présenté lors du Symposium sur l'analyse des frais tenu le 22 avril 1982 pendant le Premier Congrès International sur l'Automation en Chimie Clinique (Barcelone, Espagne)—les autres exposés ont été publiés dans le *Journal of Automatic Chemistry*, Vol. 5, No. 2.

L'auteur présente des paramètres financiers utils pour l'évaluation de l'influence de l'automation sur le laboratoire clinique, en plus il présente l'effet potentiel de l'automation sur le système des hopitaux.

**Kosten-Nutzen-Parameter bei der Auswertung der Automation**

T. M. Craig

Dies ist eine der Arbeiten die am Symposium über Kostenanalyse am 22. April 1982 während des First International Congress on Automation in Clinical Chemistry (Barcelona, Spanien) vorgetragen wurde. Die andern Arbeiten wurden bereits in der *Journal of Automatic Chemistry*, Vol. 5, No. 2, publiziert.

Der Autor stellt nützliche finanzielle Parameter für die Auswertung des Einflusses der Automation im klinischen Laboratorium vor. Zusätzlich vermittelt er einige Einsicht in die potentielle Wirkung der Automation auf das Spital-System.

---

### Abstracts of papers in Vol. 5, No. 3 (July–September 1983)

The following translations could not, unfortunately, be published in the last issue:

**Die Verwendung von Mustererkennungs-Visualisierungstechniken zur Sichtbarmachung der Daten in einem komplexen Datensatz**
M. P. Derde *et al.*
Der Artikel soll zeigen, dass Visualisierungs- und Formerkennungsmethoden (Pattern Recognition) die Untersuchung von multivariablen Daten sätzen erlauben. Das wird mit einem praktischen Beispiel, die Authentifizierung des geographischen Ursprungs von Weinen, belegt. Der Datensatz besteht aus dem Aminosäuremuster von 195 französischen Weinen verschiedener Herkunft. Die in den Daten enthaltene Information wurde mit Visualisierungsmethoden (Principal Components Analysis–Non Linear Mapping) sowie mit überwachten Methoden (Linear Discriminant Analysis) und nicht überwachten Methoden (Hierarchical Clustering) sichtbar gemacht.

Aufgrund der erhaltenen Visualisierungen konnte geschlossen werden, dass die Unterscheidung von Weinen nach ihrem Ursprung mit Hilfe des Aminosäuremusters möglich sein muss.

---

**Le contrôle de qualité de données mesurées**

L. Leisztner and P. Barna

Lorsqu'on fait des mesures analytiques deux-dimensionales, l'information analytique montre toujours des déviations non-statistiques et à cause du bruit de fond des déviations standard différentes. Une méthode pour estimer la déviation standard d'erreurs non systématiques de signaux mesurés est décrite. La méthode a été testée en simulant des mesures chromatographiques avec du bruit de fond sur le logiciel. La déviation standard créé et la déviation estimée des erreurs non systématiques ne démontraient aucune différence significative.

**Die Qualitätskontrolle gemessener Daten**
L. Leisztner and P. Barna

Bei zweidimensionalen analytischen Messungen weist die analytische Information nichtstatistische Abweichungen und wegen des Rauschens verschiedenste Standardabweichungen auf. Eine Methode zur Schätzung der Standardabweichung von Zufallsfehlern gemessener Probensignale wird beschrieben. Die Methode wurde durch Simulation verrauschter chromatografischer Messungen auf einem Computer getestet. Die erzeugte und die geschätzte Standardabweichung der Zufallsfehler zeigten keinen signifikanten Unterschied.

